# Observing APOD with the AuScope VLBI Array

**DOI:** 10.3390/s18051587

**Published:** 2018-05-16

**Authors:** Andreas Hellerschmied, Lucia McCallum, Jamie McCallum, Jing Sun, Johannes Böhm, Jianfeng Cao

**Affiliations:** 1 Department of Geodesy and Geoinformation, Technische Universität Wien, Gußhausstraße 27–29/E120.4, A-1040 Vienna, Austria; johannes.boehm@geo.tuwien.ac.at; 2 School of Physical Science, University of Tasmania, Private Bag 25, Hobart TAS 7001, Australia; lucia.mccallum@utas.edu.au (L.M.); jamie.mccallum@utas.edu.au (J.M.); 3 National Astronomic Observatory, 20A Datun Road, Beijing 100012, China; sunjing@nao.cas.cn; 4 Beijing Aerospace Control Center, 26 Beiqing Road, Beijing 100094, China; jfcao@foxmail.com

**Keywords:** VLBI, APOD, AuScope, satellite tracking

## Abstract

The possibility to observe satellites with the geodetic Very Long Baseline Interferometry (VLBI) technique is vividly discussed in the geodetic community, particularly with regard to future co-location satellite missions. The Chinese APOD-A nano satellite can be considered as a first prototype—suitable for practical observation tests—combining the techniques Satellite Laser Ranging (SLR), Global Navigation Satellite Systems (GNSS) and VLBI on a single platform in a Low Earth Orbit (LEO). Unfortunately, it has hardly been observed by VLBI, so major studies towards actual frame ties could not be performed. The main reason for the lack of observations was that VLBI observations of satellites are non-standard, and suitable observing strategies were not in place for this mission. This work now presents the first serious attempt to observe the satellite with a VLBI network over multiple passes. We introduce a series of experiments with the AuScope geodetic VLBI array which were carried out in November 2016, and describe all steps integrated in the established process chain: the experiment design and observation planning, the antenna tracking and control scheme, correlation and derivation of baseline-delays, and the data analysis yielding delay residuals on the level of 10 ns. The developed procedure chain can now serve as reference for future experiments, hopefully enabling the global VLBI network to be prepared for the next co-location satellite mission.

## 1. Introduction

The Global Geodetic Observing System (GGOS) has been established by the International Association of Geodesy (IAG) in order to satisfy the expected future requirements of science and society, which are facing increasing challenges on a changing planet. GGOS aims to establish a terrestrial reference frame with an accuracy on the level of 1 mm or better on a global scale [1]. This can only be achieved by a rigorous combination and integration of the different ground and space geodetic techniques. One of the most accurate realizations of a reference system aiming at this goal is the current International Terrestrial Reference Frame (ITRF2014) [2]. As a combination product, the ITRF makes use of the full history of observation time series of the four space geodetic techniques Very Long Baseline Interferometry (VLBI), Global Navigation Satellite Systems (GNSS), Satellite Laser Ranging (SLR), and Doppler Orbitography and Radiopositioning Integrated by Satellite (DORIS). In addition, the ITRF determination fundamentally relies on differential coordinates—called local ties—connecting the reference points of the geodetic instruments that are typically located within distances of a few hundred meters at co-location sites (for a definition of local ties, we refer to [3]). These local tie vectors are usually determined by classical terrestrial measurements (angles plus distances and leveling) or by GPS measurements. Although local ties play a critical role in the ITRF combination by linking the contributing techniques (along with Earth rotation parameters), comparisons of the local ties and differential coordinates determined by various space geodetic techniques reveal significant discrepancies with residuals on the centimeter level in many cases. This issue was discussed in detail among others by Altamimi et al. [4], Altamimi et al. [2], Seitz et al. [5], and Thaller et al. [6].

As proposed, e.g., by Rothacher et al. [7], an additional way to combine space geodetic techniques—complementary to local ties at co-located sites on the Earth’s surface—is the co-location of geodetic instrumentation on board Earth orbiting satellites. Such so-called “space ties” (in accordance with the term local tie) would provide unprecedented possibilities to connect the coordinate frames of different space geodetic techniques, and they would help to reveal unknown technique-specific biases. Alternative approaches to establish frame ties are the co-location of VLBI with other techniques on the moon [8], and the application of so-called global ties [9], i.e., of commonly estimated parameters in different technique solutions of space geodesy.

So far, there have been several successful attempts to combine the three satellite techniques GNSS, SLR, and DORIS via co-location on Earth-orbiting satellite platforms. Thaller et al. [6] demonstrated that it is feasible to combine GNSS and SLR by utilizing GPS and GLONASS satellites equipped with SLR retro-reflectors as co-location platforms in space. In a more recent study by Sosnica et al. [10], satellites of other GNSS constellations (BeiDou and Galileo) were used to establish the SLR-to-GNSS frame connection. Zoulida et al. [11] studied the combined analysis of GNSS, SLR, and DORIS on board the Jason-2 satellite with the goal to set a basis for the integration of a low Earth orbit (LEO) multi-technique satellite such as Jason-2 as space tie in the TRF determination.

Contrary to that, space ties with VLBI have not been established so far and have solely been able to be studied in simulations: Plank et al. [12] investigated general aspects such as suitable satellite orbits and antenna networks, finding that station position estimates at the level of a few millimeters in weekly solutions are achievable based on VLBI satellite observations only. Initial scheduling strategies for VLBI observations of the GNSS constellations were simulated and investigated by Plank et al. [13]. Anderson et al. [14] simulated VLBI observation, time-of-flight measurements using a time-encoded signal in the satellite transmission, and differential VLBI observations using angularly nearby quasars as calibration sources, and investigated their suitability for the estimation of frame tie parameters.

However, the actual link between the satellite techniques GNSS, SLR, and DORIS and VLBI has not yet been established. The essential problem in this respect is the absence of real satellite observations with VLBI radio telescopes. While GNSS, SLR, and DORIS are operationally applied for observations of Earth satellites, geodetic VLBI is intrinsically different, with its roots being settled in astronomy. Present applications of VLBI satellite tracking can mainly be found in the field of planetary sciences, with VLBI being used for the navigation and positioning of spacecrafts (for example: [15,16,17]). The requirements on observation schemes and data processing essentially differ between VLBI observations being carried out for the purpose of interplanetary navigation and for establishing frame ties via Earth satellites. Hence, observation and processing routines established for planetary science cannot simply be adopted for use in geodetic VLBI.

In the past few years, several groups within the geodetic community reported an effort on observing L-band GNSS satellite signals. Initial experiments were carried out to investigate basic signal chain characteristics and tracking capabilities mainly involving the European observatories in Wettzell (Germany), Onsala (Sweden), and Medicina (Italy) (described in, e.g., [18,19,20,21]). In an extensive series of experiments with observations of GNSS satellites (L1 and L2 signals) on the Australian baseline Hobart–Ceduna the complete process chain—from scheduling, actual observations, correlation and fringe fitting, to the final analysis of the derived group delays—could be closed for the first time [22].

One main challenge in these experiments was the standard equipment of legacy geodetic VLBI antennas, being restricted to observations in the S- and X-bands and not covering the L-band frequency range. However, this may change in the near future with the advent of broadband antenna feeds and matching receivers following the VGOS design concepts [23]. VGOS-compatible antenna equipment is expected to cover the frequency domain from 2 to 14 GHz. This will enable more flexibility in the selection of observation targets, though the frequency range again does not cover the L-Band of GNSS satellites. Hence, scientists had to fall back on antenna equipment that is not operationally used for geodetic applications involving the risk of introducing unexpected biases and unknown systematic effects caused by the non-standard signal chain.

Two dedicated co-location satellite missions were proposed by the geodetic community in recent years that would employ all four techniques used in the TRF combination: the Geodetic Reference Antenna in Space (GRASP [24]) and the European Geodetic Reference Antenna in Space (E-GRASP/Eratosthenes [25].) The GRASP satellite was planned to be equipped with a VLBI beacon emitting signals in the S- and X-bands that are compatible with frequency ranges of legacy and future VLBI antennas. The signals should have been encoded using Binary Offset Carrier (BOC) modulation to enable range measurements. The E-GRASP mission was proposed to carry a broadband noise beacon emitting signals in the S-, X- and Ku-bands.

Since a GRASP-like satellite has not yet been realized, alternative observation targets must be employed for hands-on observation experiments. Actual VLBI satellite tracking experiments are considered valuable for future co-location satellite missions as they provide plenty of hands-on expertise, complementing the information derived from theoretical simulation studies. Only actual tests are capable of revealing certain deficiencies on the data acquisition level, such as tracking issues, and allow for the experience required to adopt existing VLBI infrastructure components—hardware as well as software—according to the needs of satellite observations. Hence, today’s experiments—although they may not deliver highly accurate results—provide a great test bed for future missions with potentially a high impact on geodesy.

In this paper, we discuss VLBI observations of the APOD-A nano satellite (hereinafter abbreviated as APOD). APOD can be considered as the first realization of a co-location LEO satellite combining the techniques SLR, GNSS, and VLBI on one platform (details are provided in Section 2.2).

We describe a set of intensive tracking sessions to APOD using the AuScope VLBI array in Australia [26]. We focus on the data acquisition scheme for observations of a fast moving LEO satellite on regional baselines and discuss the resulting challenges for the tracking procedures and the data processing.

**Figure 1:**
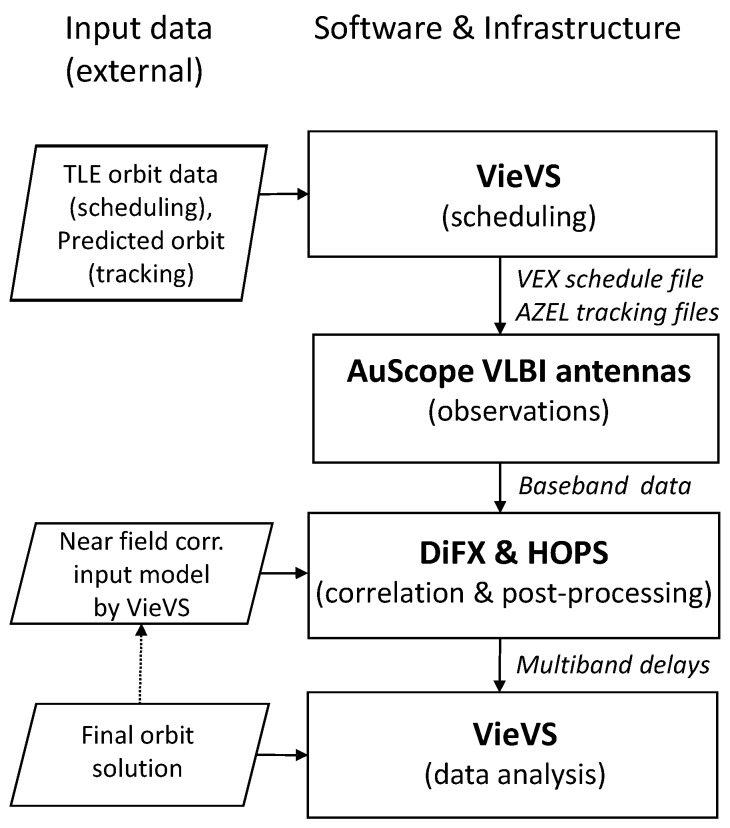
Simplified experiment work flow diagram illustrating the external input data (rhomboid boxes, left), the used software and infrastructure (rectangular boxes, right), and the essential interchange data (italic text).

The established process chain for our experiments is outlined in Figure 1. All tasks and processing steps are described in detail in the following sections. Section 2 provides an overview of all experiments discussed in this paper, and introduces the station network and the observation target. The observations, including all steps from scheduling with the Vienna VLBI and Satellite Software (VieVS [27]) to the actual data acquisition, are discussed in Section 3. In Section 4, the correlation and post-processing steps, based on the software correlator DiFX [28] and the Haystack Observatory Post-processing System (HOPS, http://www.haystack.mit.edu/tech/vlbi/hops.html), are described. The analysis of the derived delay observables using VieVS is presented in Section 5. The paper is concluded with a discussion and future prospects in Section 6.

## 2. Experiments

The presented experiments aim at deriving group delay observables from direct observations of the APOD satellite with the geodetic VLBI antennas of the AuScope VLBI array. Following initial APOD tracking tests in April and July 2016 [29] the discussed series of experiments was carried out in November 2016. A complete list is shown in Table 1. In total, we observed 8 sessions applying the experiment design described in Section 3.

### 2.1. VLBI Station Network and Tracking

**Figure 2:**
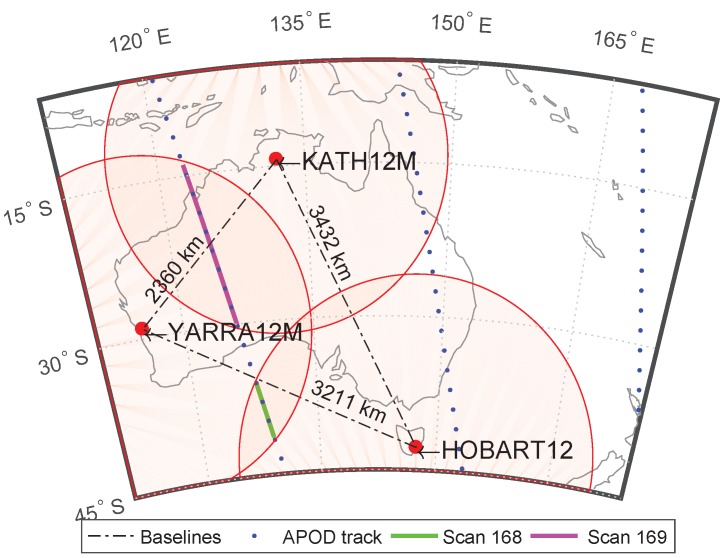
The AuScope Very Long Baseline Interferometry (VLBI) array consists of the three antennas at Hobart (Hb, Tasmania), Katherine (Ke, Northern Territory), and Yarragadee (Yg, Western Australia) indicated by filled red dots. The continental wide baselines (dotdashed lines) with lengths from 2360 to 3432 km only allow for limited mutual visibilities of the APOD satellite which orbits the Earth on an altitude of only ∼470 km. Projected fields of view of individual sites are displayed by shaded red circles assuming local cut-off elevation angles of 5${}^{{}^{\circ}}$. The APOD ground tracks during Experiment a332 (27 November 2016; see Table 1) are represented by blue dots indicating successive satellite positions in a 30 s interval. The projected APOD positions in Scans 168 (10:41:04 to 10:42:30 UTC) and 169 (10:44:02 to 10:48:18 UTC) of Experiment 332a are indicated by lines in green and magenta, respectively.

The Australian AuScope geodetic VLBI array [26] consists of three radio telescopes: HOBART12 (Hb), KATH12M (Ke), and YARRA12M (Yg). Their geographical locations and the lengths of the resulting baselines are indicated in Figure 2. The antennas are similar in design with small 12 m reflectors and fast slew rates of up to 5${}^{{}^{\circ}}$/s in azimuth and up to 1.5${}^{{}^{\circ}}$/s in elevation.

In addition to the standard tracking mode for natural sources such as quasars, Antenna Control Units (ACUs) provide a dedicated AZEL tracking mode which is applicable for satellite tracking. In this mode, an ACU loads lists of time-tagged azimuth and elevation positions and interpolates the actual pointing angles smoothly between these tracking points. In initial tests, other tracking schemes have been tested: the TLE tracking mode implemented in the NASA Field System (FS, https://lupus.gsfc.nasa.gov/fsdoc/fshome.html) and a so-called step-wise tracking approach. The latter enables satellite tracking by defining discrete source positions in terms of topocentric right ascension and declination angles in short intervals of, e.g., 1 s in the VEX file. The high position update rate in combination with the antenna’s inertia enable tracking of the satellite more or less continuously. Both schemes failed to track APOD, because they are implemented in the FS, which is simply overcharged by the high rate of positioning commands required to track such a fast satellite. In contrast, the AZEL tracking mode of the ACU directly controls the antenna without any detour via the FS, with the shortcoming that the standard control procedures for experiment automation are not accessible. Hence, manual interaction is required to switch between tracking modes and to load the AZEL tracking files individually at each antenna which is error-prone and time-consuming.

All AuScope antennas are equipped with modern Digital Base Band Converters (DBBC-2) for digitization and formatting and Mark5B+ recorder systems. The antenna feeds are designed for the S- and X-band domains and receive circular polarized signals. For more technical details, we refer to [26].

### 2.2. The APOD-A Nano Satellite

The Chinese Atmospheric density detection and Precise Orbit Determination (APOD) mission operated by the Beijing Aerospace Control Center (BACC, China) consists of a set of four CubeSats launched on 20 September 2015. It is mainly intended to carry out in-situ measurements of the atmospheric density along with the derivation of density parameters via a precisely determined orbit. For details on the scientific tasks, the payload, and the Precise Orbit Determination (POD), we refer to [29,30]. In this paper, we discuss observations of one of these satellites, named APOD-A nano, which was deployed in a near polar orbit with an inclination of about 97${}^{{}^{\circ}}$ and an initial altitude of about 470 km. Its size is only 391 × 398 × 398 mm, and it is equipped with a dual-frequency GNSS receiver (GPS and Beidou), an SLR retro-reflector, and a VLBI beacon. The VLBI beacon transmits signals in the S- and X-bands with narrow band carrier tones centered at $f_{Carr_{S}}=2262.01$ MHz and $f_{Carr_{X}}=8424.04$ MHz, respectively. Both carrier tones are symmetrically surrounded by four Differential One-way Ranging (DOR) tones designed according to recommendations of the Consultative Committee for Space Data Systems (CCSDS 401.0-B [31]). The DOR tone frequencies are calculated as $f_{DOR_{1,2}}=f_{Carr}\pm f_{Carr}/440$ and $f_{DOR_{3,4}}=f_{Carr}\pm f_{Carr}/2200$. All tones are listed in Table 2. The resulting frequency spans are 38.3 MHz in the X-band and 10.3 MHz in the S-band. The Effective Isotropic Radiated Power (EIRP) of the carrier tones is 4 dBm with the DOR tones being 12 dB weaker.

Initially, the BACC performed the POD by analyzing GPS L1 and L2 carrier phase measurements and pseudorange data with their proprietary software. As described by Sun et al. [29], the RMS of pseudorange residuals and carrier phase residuals were below 2 m and 2 cm, respectively. APOD-A was also observed by the International Laser Ranging Service (ILRS). The obtained SLR measurements were used for an independent validation of the orbit derived by GPS, by comparing SLR measurement ranges with ranges derived by GPS POD. The comparison showed good agreement with three-dimensional position deviations below 10 cm [29].

Unfortunately, the GNSS receiver partly failed in January 2016. Since then, GNSS raw-data records, i.e., pseudorange and carrier phase measurements, are no longer accessible by the ground segment. After failure, the orbit determination completely relies on three-dimensional satellite coordinates that are directly calculated by the on-board receiver and transmitted to the ground segment four times a day. As a result, the accuracy of the orbit, which is solely based on these coordinate data, dropped dramatically. For the duration of our VLBI experiments in November 2016, the orbit determination residuals were in the range of 10 to 20 m [29]. Although the APOD-A satellite was observed by the ILRS, SLR observations were only sparsely available and, therefore, could not reasonably contribute to the orbit determination.

The BACC generated and provided two kinds of orbit solutions for our APOD experiments, both solely based on the GPS coordinate data: (1) predicted orbits and (2) final orbit solutions. The predicted orbits, which were provided about 12 h before the experiments started, were used to calculate the antenna pointing data required to steer the antennas throughout the observations (see Section 3). The final orbit solutions do not contain any predictions and were calculated some weeks after our experiments took place. They were used for the calculation of a priori delays (see Section 4.1) required for the correlation, as well as for the data analysis. As mentioned above, these final orbits show post-fit residuals of 10–20 m. A comparison of all available predicted and final orbits revealed differences of several 100 m (up to 1 km) in the along-track and cross-track directions. In the radial direction, the discrepancies are much lower with values in the range of several meters. Hence, when we assume that the final orbits are accurate on a level of 10–20 m, we can conclude that the predicted orbits used for satellite tracking were only accurate on the level of several hundred meters. The rather low accuracies of the predicted as well as of the final orbit solutions have consequences on different stages of the observation and data processing schemes discussed in this paper.

## 3. Scheduling and Observations

The starting point of each VLBI experiment is the observation planning, referred to as scheduling. In general, VLBI schedules define which antennas observe which source at what time. The scheduling task itself is complicated due to the large number of different observation criteria that have to be considered, such as common visibility of the target from remote sites, slew times between consecutive scans, and the determination of required on-source time to reach the target SNR. For planning the discussed experiments, the VieVS satellite scheduling module [32] was used. It allows for the scheduling of VLBI observations of near-field targets along with observations of natural extragalactic radio sources, which are routinely observed in geodetic VLBI campaigns. The natural sources are selected automatically by optimizing the sky coverage at all stations as common for geodetic VLBI sessions [33].

The observation geometry that was determined by continental-wide baselines and the very low orbit of APOD was a major limiting factor for scheduling. On average, the APOD satellite was visible by individual AuScope telescopes four times a day for a couple of minutes only. The projected fields of view are displayed in terms of shaded red circles in Figure 2. Only in the intersecting areas was APOD simultaneously visible by two stations. As indicated in Figure 2, common visibility from all three AuScope telescopes was restricted to very low elevation angles and scan durations as short as a couple of minutes at most. In general, APOD was simultaneously visible by only two of the three antennas, limiting the experiment design to single-baseline scans. Due to these restrictions, it was not possible to observe more than two single-baseline scans shortly after another during a flyover, as exemplarily indicated in Figure 2 for two consecutive scans (168 and 169) in Experiment a332. In this example, APOD was observed first on the baseline Hb–Yg for 86 s followed by a second scan on Ke–Yg lasting for 256 s. The next occasion for an APOD scan only existed about 10.5 h later that day. Despite these circumstances, we took every chance from November 11 to 14, 2016, and observed APOD whenever common visibility from two AuScope antennas was given. All observed experiments are listed in Table 1, which also provides details on the observation durations. During observations, APOD was tracked continuously by the antennas as long as common visibility was given and the signal was recorded continuously. (Each of these recorded APOD tracks is referred to as *scan* in this paper, which must not be mixed up with the term *observation*. Due to the high SNR, delays could be derived in a 1 s interval, i.e., a single scan is chopped up into multiple observations.)

Due to the observation geometry APOD scans were restricted to low elevation angles in general with most experiments being observed at elevations between 5 and 20${}^{{}^{\circ}}$, at some rare occasions at higher elevations up to 37${}^{{}^{\circ}}$ at most. In general, the data analysis becomes more challenging due to the consistently low elevations, as the effects of the neutral atmosphere and the ionosphere on the observed signals increase with decreasing elevation. Furthermore, estimates of station heights, zenith wet delays, and station clocks do not decorrelate properly without observations at varying elevations (e.g., [34]). On the other side, the change rates of the topocentric antenna pointing directions (azimuth and elevation rates) decrease as the distance between antenna and satellite increases with lower elevation angles. Hence, the demands on the tracking and on the pointing accuracy of the antenna decrease at lower elevation angles. Therefore, the APOD satellite was easier to keep within the field of view via the observing antennas at low elevations than at high elevations.

We were able to assess the received signal live during APOD tracks by a spectrum analyzer connected to the intermediate frequency (IF) channels at the recorder racks. Our experience was that the signal amplitude became increasingly unstable at higher elevation angles, especially in the X-band due to the narrower field of view. While the S-band signals were rather stable throughout all experiments, we even experienced signal loss in the X-band occasionally at elevations above ∼30${}^{{}^{\circ}}$. We think that these tracking issues were mainly caused by the low accuracy of the predicted APOD orbits used for tracking, which show offsets to the final orbits of up to 1000 m (see Section 2.2). At an elevation of 30${}^{{}^{\circ}}$ the max. pointing error caused by an offset of 1000 m in the APOD orbit was about 4.2${}^{\prime }$. With a beam-width of about 10.2${}^{\prime }$ in the X-band, pointing errors of a few arc-minutes can already be critical. Furthermore, the internal interpolation of the tracking data in the ACU might not be accurate enough to precisely follow such a fast satellite. If there is already a pointing offset caused by the tracking data, additional inaccuracies due to a non-optimal interpolation of the data in the ACU may be enough to cause severe pointing problems in the X-band—even at moderate elevation angles.

On average five quasars were observed before and after the APOD track(s) in Experiments 316a to 319a. (For the sake of brevity, we only mention quasars when we talk about natural radio sources, as the most common type of active galactic nucleus (AGN) observed in the geodetic VLBI. To be precise, we do not exclude the possibility that other types of AGN were observed as well in the described experiments.) The main reason for the inclusion of quasars in the observation plan was to use them as calibrator sources in order to establish an initial clock model in the correlation process as outlined in Section 4.2).

Experiment a332 was designed differently: Basically, it is a geodetic 24 h VLBI session consisting of 761 three-station scans to strong quasars with a minimum flux density of 0.65 Jy. For the scheduling we optimized the sky coverage at each site in order to decorrelate estimates of station clocks, station heights, and troposphere delays. Whenever visible, APOD was observed in between the quasars, resulting in four single-baseline scans to APOD in 24 h. The first two scans (168 and 169), which are illustrated in Figure 2, are used as a generic example in this paper. This particular session design with satellite scans being embedded in quasar scans allows for additional analysis options, such as using Zenith Wet Delays (ZWDs) estimated in a quasar-only solution, to correct the APOD observations a priori (for more details, we refer to Section 5).

**Figure 3:**
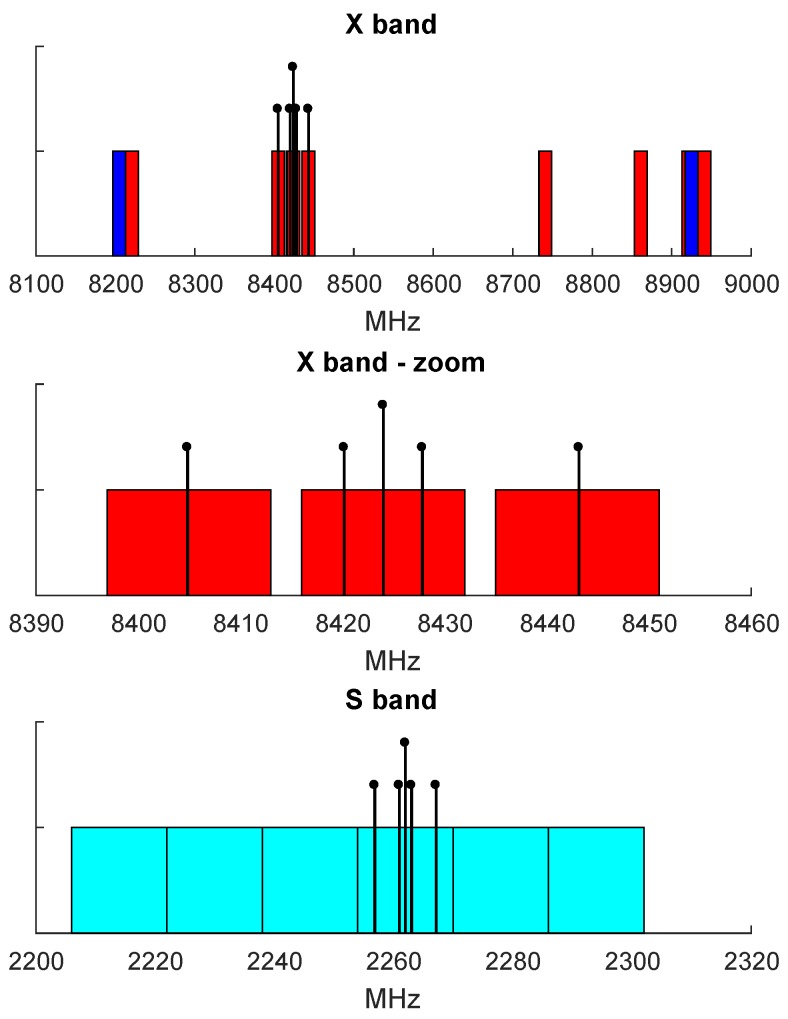
APOD signal and recording channel allocation in the frequency domain. The 16 MHz recording channels are illustrated by red (X-band, upper side band), blue (X-band, lower side band), and cyan (S-band, upper side band) boxes. The APOD tones are indicated as black lines with the carrier having a larger amplitude than the surrounding DOR tones. The X-band signals span over 38.3 MHz and were recorded in three 16 MHz channels (upper and middle panels). The S-band tones are covered by one 16 MHz channel centered at the carrier (bottom panel).

The observing mode used for all described experiments was designed pursuing two purposes: (1) to record the full APOD S- and X-band signals and (2) to derive reasonable geodetic results from standard observations of quasars at the same time. Changing the observation mode when switching between quasars and APOD was not an option as it might have introduced unknown systematic biases. The observation mode has been slightly modified from the mode used for the AUSTRAL sessions described in [35]. Data was recorded in 16 channels with 16 MHz bandwidth each, 10 in the X-band and 6 in the S-band, applying 2-bit sampling, which results in a recording rate of 64 Mbps per channel. The frequency allocation is illustrated in Figure 3. Due to strong Radio Frequency Interference (RFI) in the lower S-band at Hobart, the S-band channels were allocated contiguously, yielding continuous frequency coverage. Only 4 of 16 channels were dedicated to record the APOD tones. The purpose of the others was to increase the frequency coverage for quasar observations to enable the calculation of reasonable multi-band delays.

Publicly available two-line element (TLE) data sets (e.g., at https://celestrak.com/) were used for the initial orbit determination by VieVS and enable one to plan the experiments up to several weeks in advance. However, a final schedule iteration is recommended shortly before a session, as the observation times may slightly change with updated orbit elements. VieVS was updated for the APOD observations to generate the station-specific AZEL tracking files enabling the AuScope antennas to continuously track satellites (see Section 2.1). The data points for the tracking files were determined in a 1 s interval with the calculations being based on the latest APOD orbit predictions provided by BACC about 12 h before an experiment. The observation plans were written to VEX formatted schedule files (see http://www.vlbi.org/vex/), which are standard in VLBI. They contain a full description of the experiments, including all receiver and recorder settings for the whole antenna network, and the observation time schedule. The participating stations extract all relevant information from the VEX file using the Field System program drudg. Drudg creates station-specific control files which contain all required commands to run the experiment fully automated by the Field System, including antenna motion control, calibration routines, and the setup of the signal chain.

Only one major modification from this standard procedure was required to enable satellite tracking: Prior to satellite scans, the tracking mode of the ACUs had to be manually switched over from the star tracking mode, which is used per default for astronomical sources, to the AZEL tracking mode described in Section 2.1, and the prepared AZEL tracking files had to be loaded. These setup changes had to be done individually for all observing antennas. Manually changing the tracking mode only affected the antenna motion control, while the signal chain was still controlled by the Field System based on the setup parameters defined in the VEX file. To incorporate these additional steps, about 5 min of idling time was defined in the observation schedules prior and after APOD tracking.

The raw data were recorded with Mark5B+ recorders and were shipped to the computation cluster in Hobart for correlation and post-processing.

## 4. Correlation and Post-Processing

Our goal was to stay as close as possible to the processing scheme that is operationally applied for geodetic VLBI sessions. Therefore, we adopted the use of standard software tools for observations of the APOD satellite: the software correlator DiFX [28] and the Haystack Observatory Post-processing System (HOPS).

### 4.1. Near-Field Delays

Contrary to extragalactic radio sources, which are usually observed by the geodetic VLBI systems, Earth-orbiting satellites are situated in the Earth’s near field. As a result, standard VLBI delay models (e.g., IERS Conventions, Chapter 11 [36]) are not suitable for satellites, mainly because the assumption that incoming wave fronts are plane is not valid. In our study, we need modeled near-field delays for APOD at two stages: (1) for the correlation a priori delays—the so-called correlator input model—and (2) for the data analysis, which is discussed in Section 5.

For the delay computation, we used VieVS 3.0 [27], which is able to model near-field delays by iteratively solving the light-time equation. The implemented formalism is described in [37]. Near-field delays are calculated in the geocentric celestial reference system (GCRS), taking into account gravitational effects of bodies within our solar system. The initial ITRF2014 station positions were corrected for various tidal and non-tidal effects, as is common in VLBI processing, to obtain the most accurate values for the actual observation epochs. Earth orientation parameters (EOP), which are required for the transition from the GCRS to the TRS, were taken from the C04 time series provided by the International Earth Rotation and Reference Systems Service (IERS). Satellite positions were taken from the final APOD orbit solution provided by the satellite operator (BACC). As described earlier (Section 2.2), accuracies of the final orbit solutions were on the level of 10–20 m only. The orbit data were provided as position and velocity time series in 1 s intervals in the WGS84 system, which were then interpolated in VieVS by 9th-order Lagrange polynomials.

For the correlator input model, delays between stations and the geocenter—so-called geocentric delays—are needed rather than delays between two stations on the Earth’s surface (baseline delays). Fifth-order polynomials valid over 30 s were fitted to the time series of geocentric delays calculated in VieVS. The coefficients of the polynomials were then written to the DiFX input model files (IM-files). For more details on the practical implementation in VieVS, we refer to [22].

### 4.2. Correlation

The data were first correlated using the DiFX 2.5 correlation software [28] installed on a small computing cluster at the Mt Pleasant observatory in Hobart. A final correlation iteration was computed on the Vienna Scientific Cluster 3 (http://vsc.ac.at/systems/vsc-3/) in Vienna, Austria, using the same software components.

A first correlation of the quasar signals was made to establish the clock model using a standard approach with the HOPS package. This initial correlation used a 1 s integration time and a spectral resolution of 62.5 kHz. Yarragadee was chosen as the reference station and the a priori clock model of Hobart and Katherine were established from strong detections selected from quasar scans across the duration of the experiment.

For the APOD scans, the a priori model was inserted via modified IM-files, as described in Section 4.1. To properly handle the narrow DOR tones of the satellite, the zoom-band option in DiFX was used. This enabled the extraction of the narrow bandwidth tones of the APOD signal from the recorded 16 MHz channels (see Figure 3). Ten 32 kHz wide zoom-bands centered on the APOD DOR and carrier tones, as listed in Table 3, were used to extract the APOD signal. These scans were correlated at a fine spectral resolution of 1 kHz and a 0.1 s integration time.

Detailed investigations give us confidence on the consistency between quasar and APOD data. There are no intrinsic differences between the data. All data follows identical observing configuration with no changes to the analog pathway. Zoom-bands as implemented in DiFX do not cause any systematic differences.

**Figure 4:**
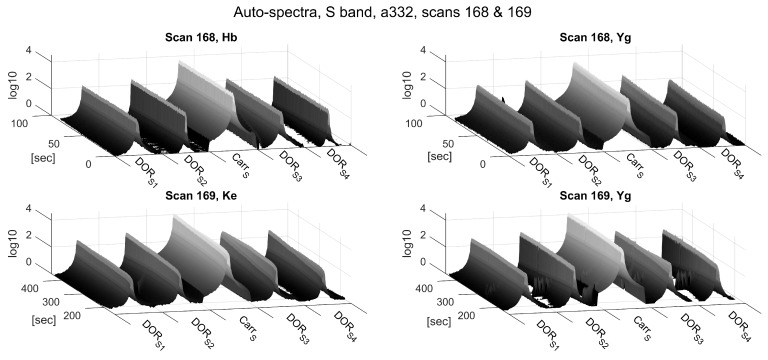
Auto-spectrum magnitudes of the S-band zoom-bands of Scans 168 (upper panels) and 169 (lower panels) of Experiment a332. The frequency axes (horizontal axes) depict the five 32 kHz wide DiFX zoom-bands described in Table 3 next to one another. The time axes indicate seconds since the start of Scan 168 (10:41:04 UTC).

**Figure 5:**
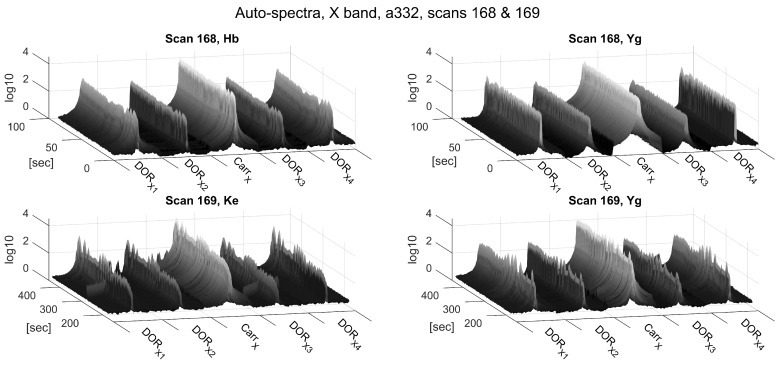
Auto-spectrum magnitudes of the X-band zoom-bands of Scans 168 (upper panels) and 169 (lower panels) of Experiment a332. The frequency axes (horizontal axes) depict the five 32 kHz wide DiFX zoom-bands described in Table 3 next to one another. The time axes indicate seconds since the start of Scan 168 (10:41:04 UTC).

Figure 4 and Figure 5 show the magnitudes of the auto-spectra of Scans 168 and 169 in Experiment a332 for all zoom-band channels in the S- and X-bands, respectively. The signal magnitudes in the S-band are much smoother and more constant over time compared to the X-band. Presumably, these perturbations are caused by tracking inaccuracies caused by the low quality of the predicted APOD orbits, which were used to calculate the tracking data, and potential deficiencies of the AZEL tracking mode of the ACU (see Section 3). In general, tracking issues have a much larger effect on the X-band, as the width of the directional receiving pattern’s main lobe, referred to as the antenna beam-width, is much narrower in the X-band. The beam-width can be approximated by BW≈λ/D, where *D* is the antenna diameter and λ denotes the wavelength of the observed signal. Hence, the beam-width in the S-band (∼38.1${}^{\prime }$) is about four times larger than in the X-band (∼10.2${}^{\prime }$), resulting in more relaxed demands on the pointing accuracy in the S-band.

**Figure 6:**
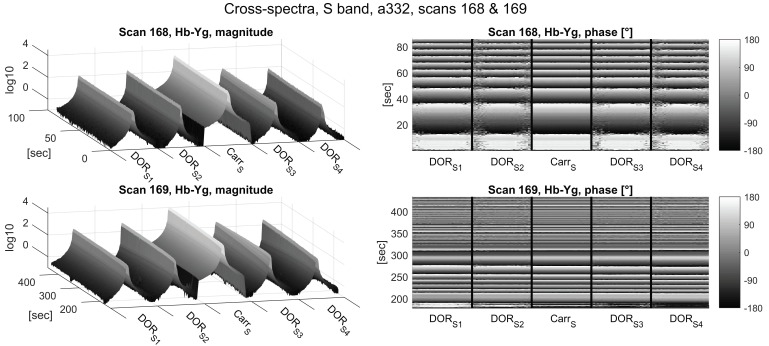
Cross-spectrum magnitudes (left column) and phases (right column) of the S-band zoom-bands of Scans 168 (upper panels) and 169 (lower panels) of Experiment a332. The frequency axes (horizontal axes) depict the five 32 kHz wide DiFX zoom-band described in Table 3 next to one another. The time axes indicate seconds since the start of Scan 168 (10:41:04 UTC).

**Figure 7:**
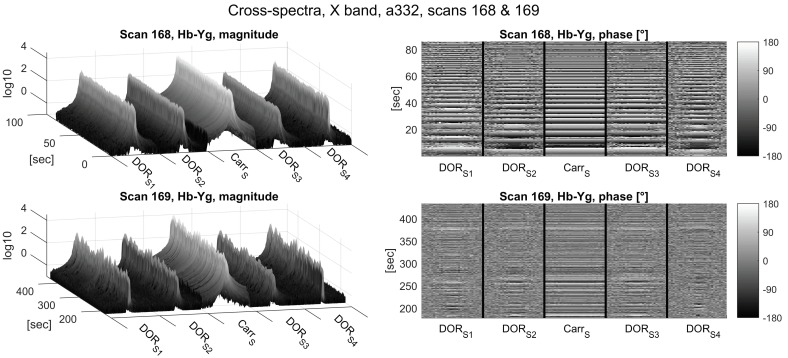
Cross-spectrum magnitudes (left column) and phases (right column) of the X-band zoom-bands of Scans 168 (upper panels) and 169 (lower panels) of Experiment a332. The frequency axes (horizontal axes) depict the five 32 kHz wide DiFX zoom-band described in Table 3 next to one another. The time axes indicate seconds since the start of Scan 168 (10:41:04 UTC).

The cross correlation spectra depicted in Figure 6 (S-band) and Figure 7 (X-band) show a similar pattern in the magnitude: again, magnitudes in the S-band are more stable over time compared to the X-band.

**Figure 8:**
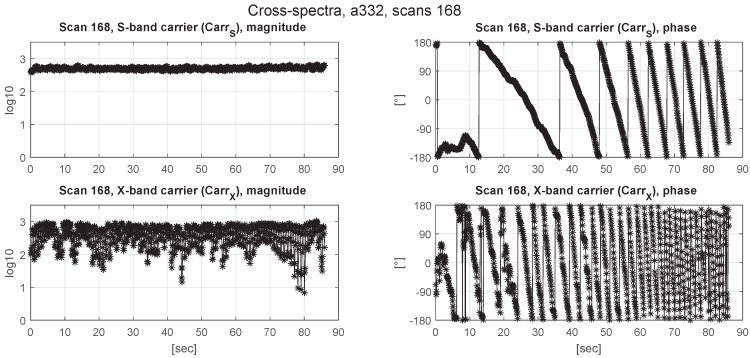
Cross-spectrum magnitudes (left column) and phases (right column) of the S-band (Carr${}_{S}$, upper panels) and X-band (Carr${}_{X}$, lower panels) carrier tones of Scan 168 of Experiment a332. The time axes (horizontal) indicate seconds since the start of Scan 168 (10:41:04 UTC).

The a priori delay model used for correlation was not accurate enough to stop phase wrapping due to residual delay rate, as evident from the phase plots in Figure 6, Figure 7 and Figure 8. Due to the smaller wavelength, the phases wrap about four times faster in the X-band, limiting the integration time to 0.1 s in the correlation process.

### 4.3. Fringe Fitting

All fringe fittings of the APOD data used the zoom-bands as an input. Across the 32 kHz zoom-band channels the APOD signal is coherent. Using the fringe fitting program fourfit (as part of HOPS), a multi-band delay was fitted for each of the two frequency bands. Due to the residual delay rate, the minimum integration time of 1 s was used to generate a time series of data.

On the initial inspection of the data, large offsets (on the order of tens to hundreds of ns) in the estimated residual delays between the S- and X-band were noted. For the frequency sequence defined by the DOR tones, we expect a multi-band delay ambiguity spacing of 972.8 ns at the S-band and 261.2 ns at the X-band. Manually applying integer multiples of these ambiguities revealed that a constant additional clock offset of −2.9 μs at Hobart and 7.8 μs at Katherine removes the large offsets between the two bands. These offsets stayed approximately the same for all experiments and are assumed to be related to the orbit modeling or the absolute timing of the observations. Another indication of this apparent ambiguity issue is found in the phase calibration, when applying a manual phase calibration in fourfit. After introducing the additional offsets, a flat residual phase against frequency is obtained. This is expected when using zoom-bands, which are taken from a common baseband converter, as is the case for all S-band channels.

**Figure 9:**
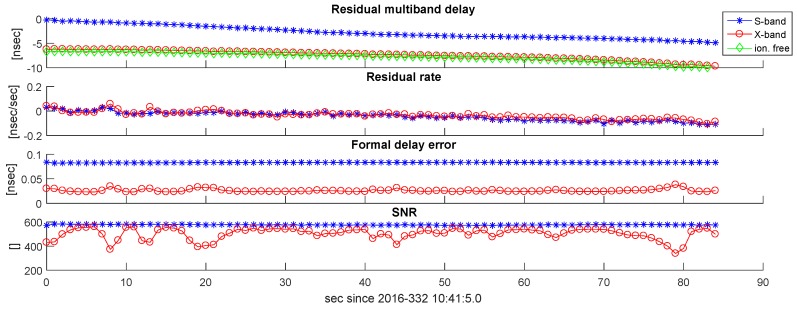
Fourfit results of Scan 168 in Experiment a332. Residual multi-band delays, residual delay rates, formal delay errors, and SNRs are plotted versus time, indicated as seconds since scan start.

**Figure 10:**
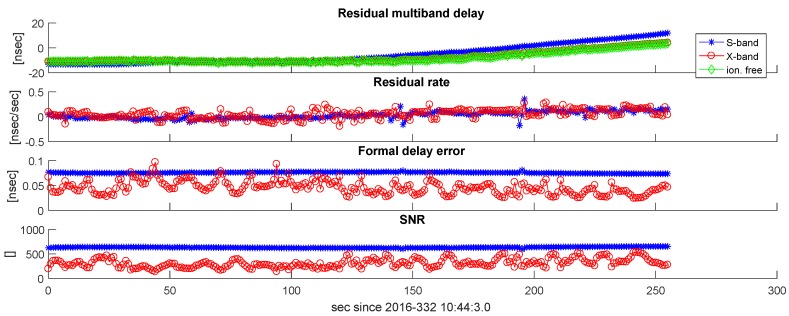
Fourfit results of Scan 169 in Experiment a332. Residual multi-band delays, residual delay rates, formal delay errors, and SNRs are plotted versus time indicated as seconds since scan start.

Examining the results of our scans shows clear differences between the S- and X-band data in amplitude, while the residual delay and delay rate are in good agreement. Examples are shown in Figure 9 and Figure 10. Signal-to-noise ratios are typically 500–800 and stable across the track for the S-band, while the X-band data show a time-variable SNR between 200 and 700. The dominant effect here is expected to be mis-pointing of the antennas. The formal error for the S- and X-bands is typically less than 100 ps.

The observations in two frequency bands, S and X, enabled us to correct for dispersive propagation effects of the ionosphere on the observed microwave signals. First-order effects are accounted for by calculating the ionosphere free linear combination according to Equation (1) [38]. The ionosphere free delays $\tau_{if}$ were determined as linear combination of the observed group delays in the S- and X-bands, $\tau_{gs}$ and $\tau_{gx}$, respectively. The factors c1 and c2 are functions of the effective frequencies of the observed S- and X-band channels, $f_{gs}=2256.85$ MHz and $f_{gx}=8404.87$ MHz.

(1) $$\begin{array}{cc} \tau_{if} & =c1\cdot \tau_{gx}-c2\cdot \tau_{gs}\;\mathrm{with}\\ c1 & =\frac{f_{gx}^{2}}{f_{gx}^{2}-f_{gs}^{2}}=1.0777\\ c2 & =\frac{f_{gs}^{2}}{f_{gx}^{2}-f_{gs}^{2}}=0.0777 \end{array}$$

## 5. Data Analysis

The multi-band delay observables derived by the processing scheme outlined in Section 4 were analyzed with VieVS 3.0., which is able to handle quasar observations, as well as observations of satellites. All target parameters can be estimated in a least-squares adjustment (Gauss–Markov model, e.g., [39]) based on reduced observations, which are calculated by subtracting the computed (theoretical) delays from the observed delays (observed minus computed, o-c).Theoretical delays for APOD observations were calculated with the near-field delay model outlined in Section 4.1 using the final orbit solution by BACC (see Section 2.2).

The analysis is based on the so-called total delays, which were computed as the sum of multi-band delay residuals calculated in fourfit and the a priori delays of the correlator input model. The observation reference epochs are the times of the signal reception at Station 1 (of the baseline), calculated at integer seconds, as common in geodetic processing. Basically, we have three sets of delay results for each experiment: (1) S-band delays, (2) X-band delays, and (3) the ionosphere free linear combination. To minimize the effect of the ionosphere, we decided to use Option 3, although the quality of the S-band observations—in terms of amplitude stability and SNR (see Section 4)—seems to be higher.

**Figure 11:**
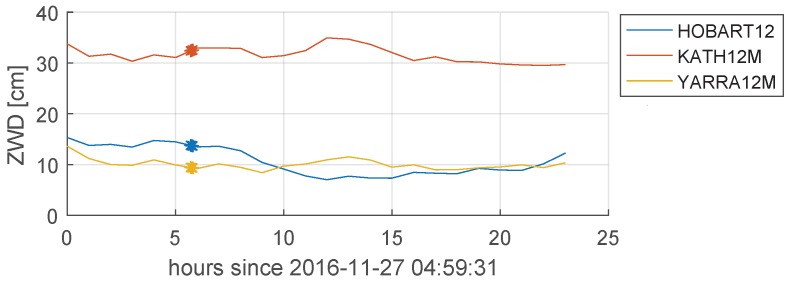
Zenith Wet Delay (ZWD) estimated from quasar observations in Experiment a332. The time at which the APOD Scans 168 and 169 were observed are highlighted.

Multi-band delays were also obtained from the quasars observed in Experiment a332 by standard VLBI data processing with DiFX and fourfit. These quasar delays were written to an Mk3 database, processed in νSolve [40] and exported as a Level 4 NGS file. They were then analyzed in VieVS by estimating a set of standard parameters: station coordinates, source coordinates, EOP, ZWD, and station clock parameters. The WRMS of the post-fit residuals was 38 ps. The obtained ZWD estimates shown in Figure 11 were then applied a priori on the APOD observations to correct for effects caused by the wet fraction of the atmosphere. The Vienna Mapping Function (VMF1 [41]) was used to determine the slant delay corrections.

**Figure 12:**
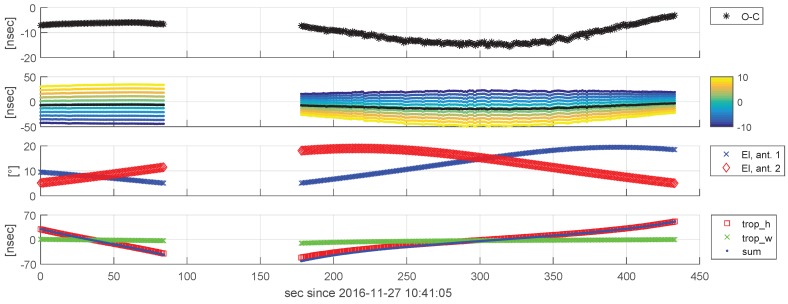
Residual delays (o-c) and modeled delay corrections of Scans 168 and 169 of Experiment a332. From top to bottom: (1) observed delays (ionosphere free linear combination) minus computed delays (o-c), (2) o-c residuals with the APOD position shifted by values between −10 and +10 m in the along-track direction (shift amounts are color-coded, no shift indicated by black line), (3) observation elevations at Stations 1 (blue crosses) and 2 (red diamonds), and (4) applied corrections for the hydrostatic (red squares) and wet parts (green crosses) of the atmosphere and the sum of both (blue dots).

Investigating the o-c delay residuals provide a first impression of how well the observed delays match the theoretical delays modeled in VieVS. The top panel in Figure 12 depicts o-c values for two consecutive APOD scans, 168 and 169, in Experiment a332. The observed delays were corrected for the station clock offsets and rates applied in the correlation step to keep the absolute o-c values small.

The bottom panel in Figure 12 depicts the major delay corrections applied on the purely geometric near-field delays (see Section 4.1), which target the effects of the hydrostatic (trop_h) and the wet (trop_w) fraction of the troposphere. The hydrostatic part was modeled according to [42] using in situ pressure measurements and the VMF1${}_{h}$ mapping function. The wet fraction was modeled by mapping the ZWD estimates derived by the quasar observations in Experiment a332 down to slant directions using the VMF1${}_{w}$ mapping function. The total troposphere corrections are in the range between ∼−61 ns and ∼+51 ns. Other corrections not being illustrated here due to their comparably small magnitudes are thermal antenna deformation (max. 1.8 ps [43]), gravitational effects on the near-field delays according to [37] (max. ±24 ps) and the effect of antenna axes offsets (max. 44 ps).

In Scan 168, the residual delays show little variation of ∼1.5 ns over the scan duration. In contrast, the delay residuals in Scan 169 are noisier and they show a systematically curved variation with a magnitude of ∼12 ns over the scan. Looking at the elevation angles at which APOD was observed during these scans (Figure 12, third panel), we suppose that the more pronounced scatter in the second scan can be explained by tracking issues that are more likely to occur at higher elevations. At first glance, the distinct bent shape in Scan 169 can lead to the assumption that this is caused by elevation-dependent effects, such as troposphere delays. However, investigations showed that this signature can be explained by a constant offset in the satellite’s along-track position present in the a priori orbit data that were used to determine the theoretical delays. The second panel in Figure 12 depicts the o-c time series derived by the same observation data, but with different constant along-track offsets (from +10 to −10 m) applied when modeling the computed delays. Typically, the largest uncertainty in orbit determination is present in the along-track direction, especially in the case of LEO satellites due to the predominant effect of the atmospheric drag, which is constantly decelerating the satellite (e.g., [44,45]). Considering the low quality of the used orbit data (see Section 2.2), deviations of 10 m (or even more) along-track can be expected. When applying a constant along-track offset of about −8 m on the a priori orbit, the bent shape disappears. The remaining constant offsets between the two scans can be explained by uncorrected clocks.

Such o-c values could in principle be used to estimate all parameters that are usually determined by the geodetic VLBI technique, plus satellite orbit parameters. Due to the severe inaccuracies in the a priori orbit data, it is mandatory to estimate orbit parameters. Otherwise, unmodeled orbit errors would propagate into other estimates. Basically, VieVS provides features to estimate satellite position offsets in terms of piece-wise linear functions, which refers to a kinematic orbit modeling approach. The presented APOD observations were used to validate and test these features. However, for kinematic orbit modeling, the available VLBI observations are insufficient. Firstly, the total number of available tracks is by far too low. Secondly, a global tracking network would be required, rather than a regional one, to acquire observations distributed over whole orbit arcs. A global network would also yield more observations in total.

## 6. Summary and Outlook

The Chinese APOD-A nano satellite can be considered as the first prototype of a co-location mission enabling VLBI with SLR and GNSS on an LEO satellite.Unfortunately, it has hardly been observed by VLBI, so major studies towards the actual frame ties could not be performed. The main reason for the lack of observations was that VLBI observations of satellites are non-standard, and suitable observing strategies were not in place for this mission. This work now presents the first serious attempt to observe the satellite with a VLBI network over multiple passes. We discuss a series of APOD observations with the AuScope geodetic VLBI array carried out in November 2016. We describe all steps—from the initial experiment design, to the observation and data acquisition procedures, to the correlation and post-processing scheme, and to the analysis of the derived delay observables in VieVS. By the example of Experiment a332, we discussed all applied processing steps, yielding o-c residuals on the level of a few nanoseconds.

One major limitation was the bad quality of the APOD orbit solutions due to the absence of GNSS raw data caused by malfunction of the on-board receiver. The predicted orbit data used for tracking show offsets of up to 1000 m compared to the final orbit solutions, which were used to calculate theoretical near-field delays for correlation and analysis. We assume that the low quality of the orbit data, in combination with potential deficiencies of the AZEL tracking mode provided by the antenna controllers, lead to pointing inaccuracies on the level of a few arc-minutes. The mis-pointing caused wide amplitude variations in the received X-band signals, resulting in a lower and more variable SNR of the X-band data compared to the S-band (where the antenna field of view is about four times wider). On the data processing level, the low quality of the a priori orbit limited the accuracy of the computed theoretical delays. Hence, the correlator input model was not accurate enough to fully stop phase wrapping. However, the delay model was sufficient to decrease the phase rate to such an extent that a practicable integration time could be used without introducing decorrelation.

Facing the problems caused by the inaccurate orbit solutions, it would be beneficial to use SLR observations (in addition to the available GNSS measurements) to improve the orbit determination. SLR measurements could not only be used to calibrate orbit errors in radial direction but also along-track errors due to the low elevation angles at which most of the LEO tracking data are collected. According to Arnold et al. [46], SLR can contribute along-track corrections on the millimeter level and thus substantially improve the overall orbit accuracy. Unfortunately, SLR measurements were sparsely available at the times VLBI observations were performed, precluding this option. It is highly advisable that, for future experiments, VLBI observations are coordinated with SLR tracking campaigns.

For the sake of brevity, only one experiment is discussed in detail. Experiment a332 was chosen as a representative example. However, delay observables could be obtained from all experiments using the presented process chain. (Results in terms of total multi-band delays for all sessions listed in Table 1 are publicly available in the IVS working group 7 Wiki at http://auscope.phys.utas.edu.au/opswiki/doku.php?id=wg7:apod:apod_obs_auscope. In addition to the results, all session control files, e.g., VEX schedules, are provided along with operator notes.) All residual multi-band delays show similar characteristics in terms of magnitude, scatter (S-band is always smoother than X-band), and SNR (higher and more stable in the S-band). Presumably, systematic signatures in the delay residuals were mainly caused by errors in the a priori orbits used to model the computed delays. Additionally, the described tracking issues could have caused small systematic effects, as the satellite beacon was observed with slight off-axis angles. In general, the delay observables become noisier with increasing elevation, especially in the X-band, indicating that improved tracking features utilizing more accurate orbit data would be beneficial.

Considering the experience gained through the presented experiments, we provide some suggestions for future experiments. In general, the common visibility of APOD was limited to elevations lower than 40${}^{{}^{\circ}}$ due to the observation geometry, which was determined by continental-wide baselines (between 2360 and 3432 km long) and the low satellite orbit (about 450 km). Shorter baselines would enable one to observe longer tracks with a larger variation in the elevation angle. Nevertheless, a global distribution of observation sites is a prerequisite for the estimation of satellite orbits. Further (simulation) studies are recommended to investigate suitable observation networks for LEO satellites.

The VLBI signal emitted by APOD, consisting of narrow band carriers plus symmetric DOR tones at redundant spacing, is not ideal for obtaining delay observables as common in the geodetic VLBI, as it yields a very narrow ambiguity spacing. For classical VLBI processing, wide band noise would be preferable, although the present signal is suitable for other processing schemes, such as Doppler ranging.

Furthermore, our experiments clearly showed that adequate satellite tracking features, which enable the antennas to track continuously and accurately are a basic requirement for observations of LEO satellites. It is important to consider this when designing the future global VLBI network in the view of co-location satellite missions.

The next step towards fully automated satellite observations is the integration of the AZEL tracking mode provided by the ACU in the Field System (station-specific code). In combination with the full support of the new VEX2.0 format (https://safe.nrao.edu/wiki/bin/view/VLBA/Vex2), which will enable one to integrate satellites in the observation schedules and control files, satellites could then be observed like quasars in geodetic sessions.

Our work showed that a sophisticated dynamic orbit model is highly beneficial for the analysis of future experiments. Considering a potentially low number of observed tracks—caused by the restricting observation geometry for LEO satellites—and a non-ideal distribution of stations, a kinematic orbit model is not suitable. To be prepared, we plan to upgrade VieVS with a suitable orbit model.

Although the results of this study are not suitable yet to study actual frame ties, this work is valuable due to the gained experience in terms of observation, data processing, and analysis strategies. The developed observation and data processing procedures can now serve as guidelines for other observatories, hopefully better preparing the global VLBI network for the next co-location satellite mission.

## Figures and Tables

**Table 1:** A list of APOD experiments by AuScope in November 2016. Experiments 316a to 319a lasted for about 40 min at most observing one or two consecutive single-baseline scans to APOD shortly after another. Experiment a332 lasted for 24 h mostly observing quasars in a geodetic schedule plus four APOD tracks in between. The last column (APOD tracks) indicates the number of APOD tracks observed in this session and the duration of each scan in seconds (in brackets).

Experiment Name	Stations	Exper. Start [UTC]	Duration (Duration [s])	APOD Tracks
316a	Ke, Yg	11 November 2016, 22:15:00	33 min 11 s	1 (211)
317a	Ke, Hb	12 November 2016, 09:34:00	41 min 33 s	1 (27)
317b	Ke, Hb, Yg	12 November 2016, 21:35:00	34 min 49 s	2 (161, 137)
318b	Ke, Hb, Yg	13 November 2016, 10:48:30	25 min 57 s	2 (41, 241)
318c	Ke, Hb	13 November 2016, 21:01:00	25 min 59 s	1 (44)
318d	Ke, Yg	13 November 2016, 22:35:00	22 min 55 s	1 (153)
319a	Ke, Hb, Yg	14 November 2016, 10:00:00	40 min 5 s	2 (74, 201)
a332	Ke, Hb, Yg	27 November 2016, 16:00:00	24 h	4 (86, 256, 29, 189)

**Table 2:** Carrier and Differential One-way Ranging (DOR) tone frequencies of the APOD VLBI signal in the S- and X-band.

Label	Band	Frequency [MHz]
Carr${}_{S}$	S	2262.010
DOR${}_{S1}$	S	2256.869
DOR${}_{S2}$	S	2260.982
DOR${}_{S3}$	S	2263.038
DOR${}_{S4}$	S	2267.151
Carr${}_{X}$	X	8424.040
DOR${}_{X1}$	X	8404.894
DOR${}_{X2}$	X	8420.211
DOR${}_{X3}$	X	8427.869
DOR${}_{X4}$	X	8443.186

**Table 3:** List of the zoom-bands specified in DiFX for the correlation of APOD observations. The 32 kHz zoom-bands are centered on the APOD VLBI tones listed in Table 2.

Label	Band	Lower Band Edge	Bandwidth
DOR${}_{S1}$	S	2256.852 MHz	32 kHz
DOR${}_{S2}$	S	2260.964 MHz	32 kHz
Carr${}_{S}$	S	2261.992 MHz	32 kHz
DOR${}_{S3}$	S	2263.020 MHz	32 kHz
DOR${}_{S4}$	S	2267.133 MHz	32 kHz
DOR${}_{X1}$	X	8404.870 MHz	32 kHz
DOR${}_{X2}$	X	8420.186 MHz	32 kHz
Carr${}_{X}$	X	8424.015 MHz	32 kHz
DOR${}_{X3}$	X	8427.845 MHz	32 kHz
DOR${}_{X4}$	X	8443.161 MHz	32 kHz
